# CMV in the gut: a critical review of CMV detection in the immunocompetent host with colitis

**DOI:** 10.1007/s10096-014-2212-x

**Published:** 2014-08-06

**Authors:** A. L. Goodman, C. D. Murray, J. Watkins, P. D. Griffiths, D. P. Webster

**Affiliations:** 1 Department of Infection and Immunity, University College London, Cruciform Building, Gower Street, London, WC1E 6BT UK; 2 Centre for Virology, University College London Medical School, Rowland Hill Street, London, NW3 2PF UK; 3 Department of Gastroenterology, Royal Free London NHS Foundation Trust, London, NW3 2QG UK; 4 Department of Cellular Pathology, Royal Free London NHS Foundation Trust, London, NW3 2QG UK

## Abstract

As scientific techniques for the detection of cytomegalovirus (CMV) improve, we are able to detect small amounts of CMV in the mucosal wall. As clinicians, we are unsure how to interpret the results of this novel test. There is controversy in the literature as to the significance of the detection of CMV in the gut. Whilst the importance of CMV and reactivation of the virus is clear in those patients such as allograft recipients with established immune compromise, the role is less clear in patients with less damaged immune systems. We explore whether the detection of CMV in such cases influences outcome and how it should be optimally managed. We discuss the optimal management of such cases, according to current guidelines, with a review of the literature.

## Introduction

In the apparently immunocompetent patient presenting with bloody diarrhoea for the first time, there is often diagnostic uncertainty as to whether the cause of colitis is likely to be infectious or inflammatory. Risk factors for an infectious aetiology include undiagnosed immune compromise [such as human immunodeficiency virus (HIV)], travel, exposure to antibiotics or hospital, and unprotected sexual intercourse. Even when an infectious colitis is diagnosed, this does not exclude the possibility of a subsequent diagnosis of an inflammatory bowel disease, which may present initially as an infective colitis. In a first episode of severe colitis which fails to respond to steroids or other immunosuppressive therapy, guidelines suggest that we should look for cytomegalovirus (CMV) at sigmoidoscopy [1]; however it is acknowledged that the subsequent detection of CMV can be difficult to interpret. The diagnosis of CMV colitis in the immunocompetent host is rare, but needs to be considered, alongside the possibility of undiagnosed immunocompromise, in the patient that fails to respond to maximal immunosuppressive therapy. Case reports and case series of immunocompetent patients with CMV colitis have been published; however, the patients included often had comorbidities or pregnancy, which could affect their immune status [2]. Although CMV may be detected at diagnosis in an immunocompetent patient with ulcerative colitis, it is more commonly diagnosed following immunosuppressive treatment for colitis. The literature regarding the importance of the positive CMV result on biopsy is reviewed here with respect to the following questions:

### Is CMV ever the sole cause of colitis in an immunocompetent host?

CMV is a major cause of morbidity in immunosuppressed patients, causing significant disease in transplant patients and, prior to the introduction of highly active antiretroviral therapy (HAART), in HIV. In such patients, end-organ involvement following viraemic spread of CMV may lead to damage to a single organ, as seen in, for example, colitis, retinitis or severe pneumonitis. CMV is also known as human herpesvirus 5 and, like other herpesviruses, it causes a primary infection followed by the establishment of latency, a dormant infection in which only a few genes are expressed. Recurrent disease can occur if the virus reactivates due to perturbations in immunity, e.g. as seen with age or immunosuppressive drugs. CMV is common, with a seroprevalence (CMV IgG-positive) of 40–100 % in adults, increasing with age [3]. Primary infection is often asymptomatic in the immunocompetent host but can cause a mild febrile illness and an infectious mononucleosis syndrome. However, single-organ pathology, such as hepatitis, retinitis or colitis, occurs rarely following primary infection or reactivation in an immunocompetent host [4].

CMV colitis in the immunocompetent patient is uncommon, though it has been described as presenting with a syndrome incorporating symptoms of colitis (e.g. abdominal pain, fever, diarrhoea, rectal bleeding). Galiatsatos et al. reviewed the literature and found 44 immunocompetent patients with CMV colitis; however, 34 of these patients had comorbidities that would be expected to affect immune function (pregnancy, renal disease, diabetes, malignancy) [2]. In that study, age over 55 years was found to be associated with a poor outcome. This small study suggested that the diagnosis in an immunocompetent patient is rare and raises the question of either an alternative diagnosis, such as a new presentation of inflammatory bowel disease (IBD), or a previously undetected immune deficiency (such as HIV). Even mild immunosuppression, as seen in chronic kidney disease, seems to predispose to CMV reactivation and colitis [5]. Therefore, the answer to this question is probably yes, but rarely.

### How can we diagnose CMV colitis?

Although a wide range of diagnostic tests for CMV is available, each has limitations (Table 1). Serology is useful to establish evidence of previous infection (CMV IgG) and IgG avidity can help to estimate the time of primary infection, as the IgM antibody can remain positive for up to a year following primary infection.

**Table 1:** Summary of diagnostic tests for cytomegalovirus (CMV)

	IgG	IgM	Avidity	Blood detection CMV (DNA)	Virus culture from urine or throat	Immunohistochemistry	Histology	CMV DNA on biopsy
Active infection	+	+/−	High/Low	+	+	+	+/−	+
Infection within last 2–4 months	+	+	Low	+/−	+/−	+/−	+/−	+/−
Infection within last 4–24 months	+	+/−	High	+/−	+/−	+/−	+/−	+/−
Inactive Infection	+	−	High	−	−	−	−	−
Reactivation	+	+	High	+/−	+/−	+/−	+/−	+/−
Reexposure	+	+	High	+/−	+/−	+/−	+/−	+/−

**Fig. 1:**
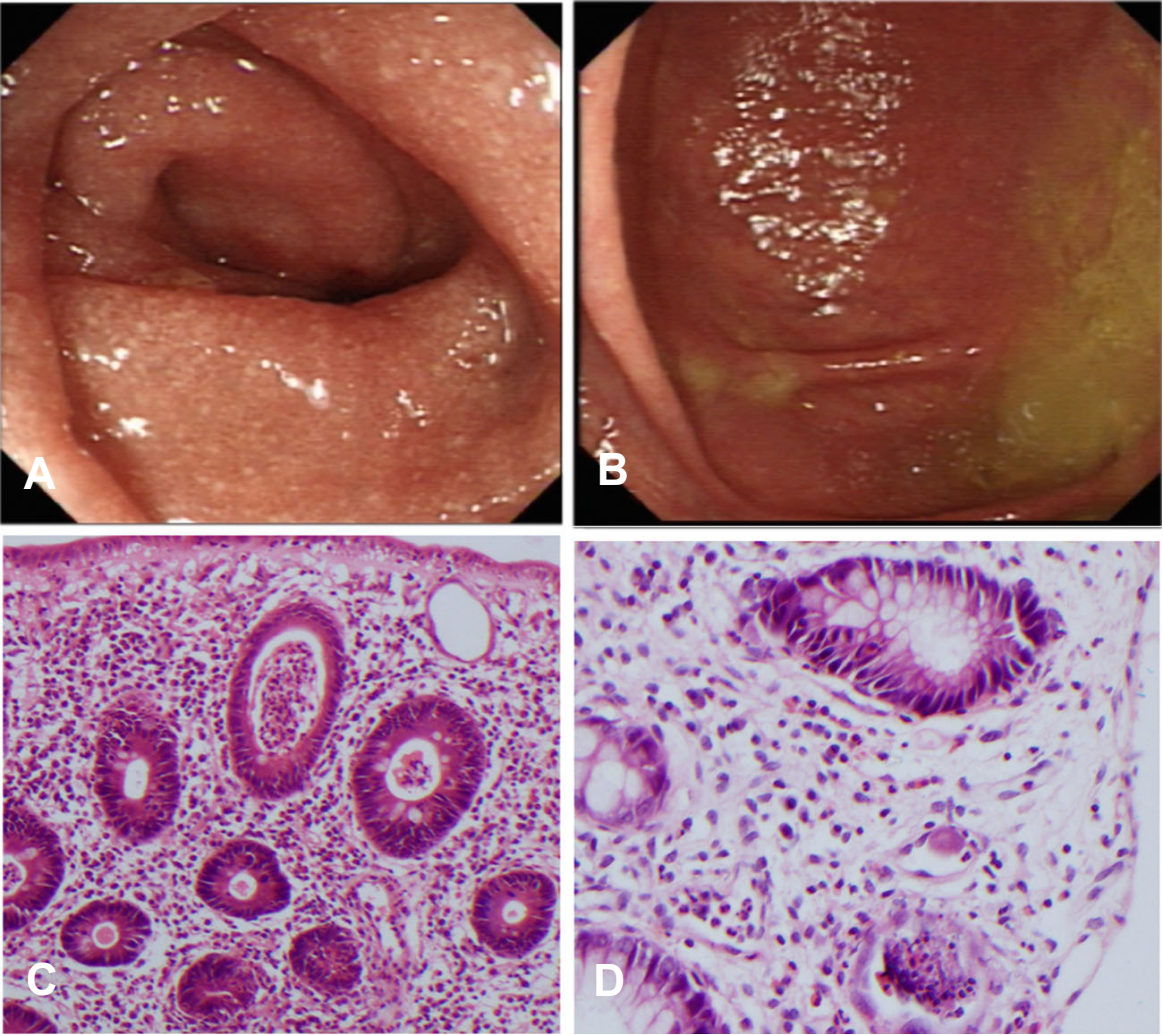
**a** and **b** show endoscopic appearances of the proximal ascending colon in our patient. **c** shows her histology and **d** shows histology from a patient with cytomegalovirus (CMV) colitis and typical owl’s eye inclusion

If CMV colitis is suspected, the bowel is examined endoscopically for evidence of CMV disease. This may be detected as typical findings on histology, such as owl’s eye inclusion bodies. This histological appearance is very specific for CMV, has a clear relation to polymerase chain reaction (PCR) detection of CMV in the gut [6] and provides the mainstay of diagnosing CMV end-organ disease post-transplant [7]. However, histology has a low sensitivity, so CMV infection may be missed. Immunohistochemistry (IHC) or simple haematoxylin and eosin (H&E) staining can be used to improve sensitivity if a diagnosis of CMV colitis is considered (Fig. 1). Histology, H&E and IHC stains retain specificity for CMV disease. CMV DNA detection in the blood by PCR has replaced the previous technique of CMV pp65 antigen detection and high levels of CMV DNA in the blood correlate with positive IHC and detection in tissue [8]. However, 15 % of people with end-organ damage causing retinitis were found to have no CMV detectable in their blood in the pre-HAART era, suggesting that viraemia does not always persist until the time of clinical presentation [9]. Modern studies suggest that CMV PCR in stool may, in due course, allow us to detect CMV with a non-invasive test [10]. Unfortunately, currently, the most sensitive test for CMV, CMV biopsy PCR, does not seem to have sufficient specificity to be used alone to make a diagnosis of CMV colitis. CMV viral cell culture is also sensitive but labour-intensive and has, therefore, been replaced by nucleic acid detection. As these tests detect CMV at very low levels, they also detect small amounts of reactivating virus that may not be causing disease. In the immunocompetent host, periodic reactivation of CMV does not necessarily indicate pathology. The significance of such small amounts of CMV in the colon has not been established. Lawlor and Moss found that the majority of published studies detected CMV DNA in the bowel in a significant proportion of patients without detection by H&E or IHC [11]. They suggest that the use of a ‘cut-off’ DNA level might aid diagnosis where CMV in the colon could be considered important only when present at above a certain level. That level has not yet been established and further work is clearly needed in this area.

### Does the presence of CMV correlate with disease activity?

**Fig. 2:**
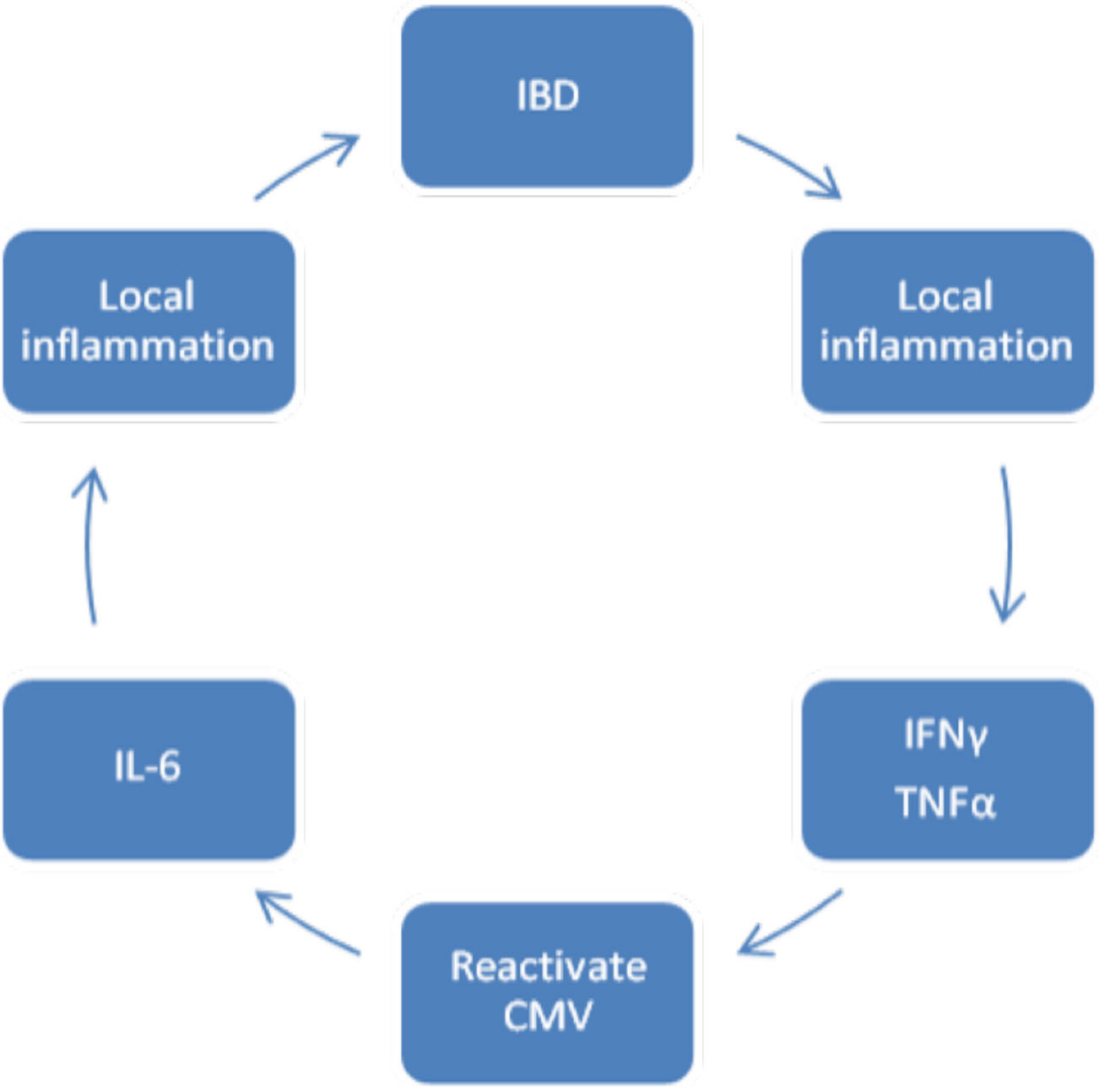
Proposed cycle of pathology

CMV has been found in approximately a third of samples from patients with severe ulcerative colitis [12]. In contrast, in inactive colitis, the detection of CMV DNA is rare [13]. Although CMV DNA is detected more often in severe colitis, it is unclear whether it is a causative factor. Alternatively, the CMV DNA may be detected because of inflammation and loss of integrity of the bowel wall. Local inflammation in the bowel wall leads to the release of cytokines such as TNF-α and IFN-γ. These cytokines may activate CMV replication and promote the migration of CMV-infected macrophages to inflamed tissue to further propagate infection (Fig. 2) [14]. If CMV was a significant factor in pathology, one would expect those patients with IBD and positive CMV IgG to have a worse outlook than patients with negative CMV IgG, but in a study of 187 patients, the two groups were found to have similar appearances of mucosa at colonoscopy [15]. In addition, the presence of CMV DNA in blood does not seem to predict the deterioration of IBD in prospective studies.

### Is CMV a precipitant for IBD?

Although the detection of CMV has been found to be associated with severe disease in IBD, there is no evidence that this relationship is causative. CMV at presentation of ulcerative colitis or IBD does occur, but is rare. Blood PCR is often positive after 2–3 weeks of steroid treatment but this rarely leads to CMV disease on histology with DNA levels lower than those seen in transplant patients and CMV DNA levels falling as steroids are reduced [16]. In transplant patients, the administration of steroids reduces the CMV viral load required to cause end-organ disease and perhaps this interaction also occurs in the immunocompetent patient [17].

### Does the presence of CMV simply reflect the degree of immunosuppression?

CMV is detected more often in cases of steroid-refractory disease [13]. It is unclear if such patients would have more CMV because they have more inflammation or because they have received greater doses of immunosuppression in an effort to control their colitis. In some patients, CMV detection occurs prior to the prescription of immunosuppressants, but in IBD, CMV is commonly detected following immunosuppressant therapy. Matsuoka et al. demonstrated a worse prognosis in those with high levels of CMV in blood leading to greater requirements for ciclosporin [16]. In turn, patients treated with ciclosporin also develop more CMV and have a poorer prognosis [18]. Studies in organ transplant recipients have shown that higher levels of immunosuppression lead to higher rates of CMV disease. Extrapolated to IBD, this suggests that more CMV may be seen when more immunosuppressants are used [19]. In the case of the detection of CMV DNA in the mucosal wall in the presence of colitis, it is usually unclear whether the CMV is the cause of pathology or an innocent bystander.

### Can treating CMV improve outcome in patients with colitis?

Although guidelines are universal in recommending the treatment of CMV in severe refractory colitis with CMV detected, this guidance is based on a number of small studies with inconclusive outcomes. A randomised controlled study in HIV in the pre-HAART era showed a benefit of treating CMV colitis in acquired immunodeficiency syndrome (AIDS) [20], but no randomised controlled studies have been done in immunocompetent hosts. Those retrospective studies or observational studies performed in immunocompetent hosts are confounded, as treatment with ganciclovir is more often used in patients with more severe disease. It is known that, in the absence of anti-CMV treatment in colitis, reactivated CMV may resolve spontaneously [16]. CMV viraemia post-transplant also frequently resolves without specific anti-CMV treatment [21]. There are insufficient published data to determine if treating CMV impacts upon major outcomes such as colectomy rates and mortality [11].

### How should we manage a patient with CMV detected on colonic biopsy?

**Fig. 3:**
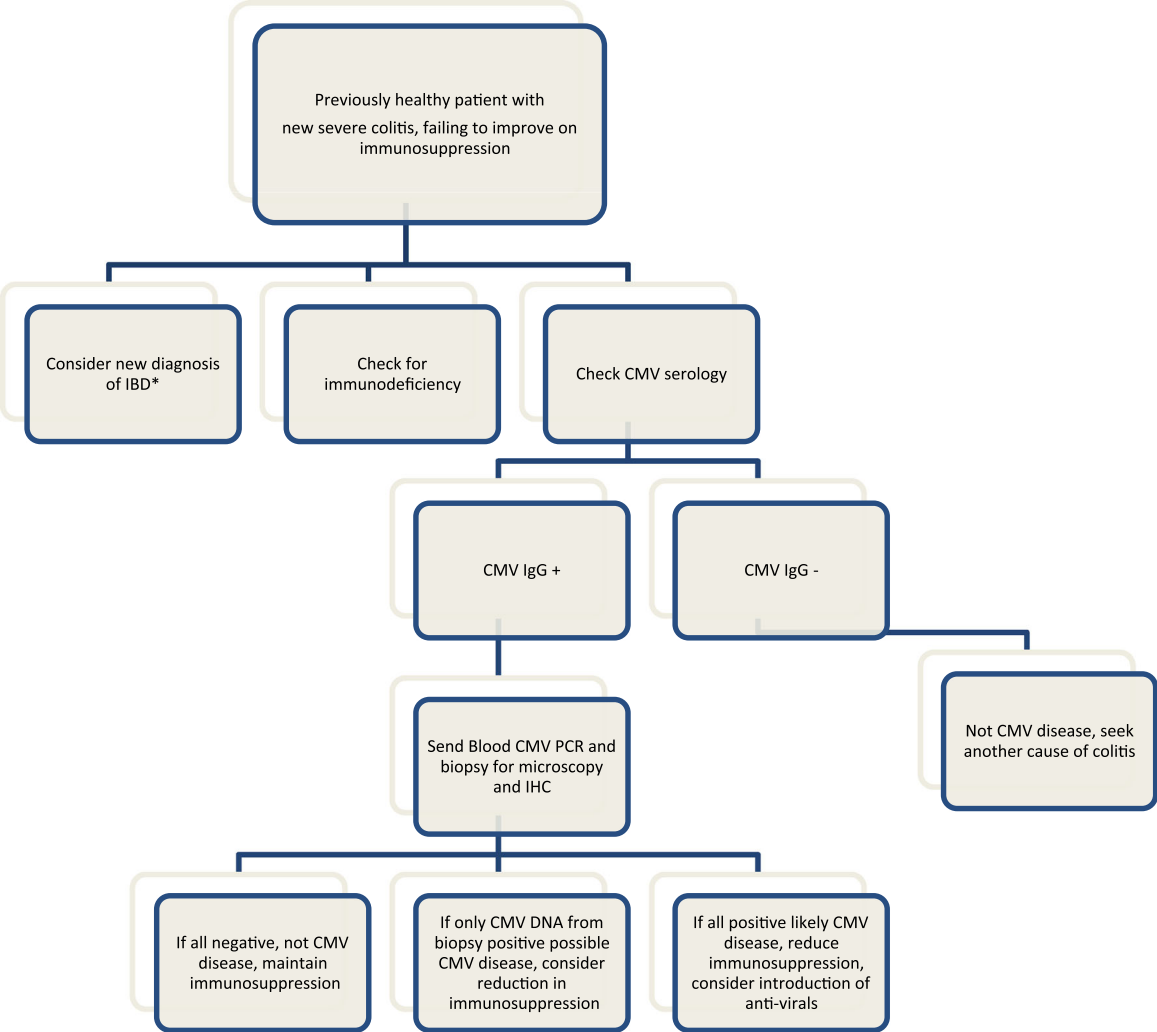
Flow chart of suggested management

The American College of Gastroenterology (ACG) and the European Crohn’s and Colitis Organization (ECCO) recommend treatment of CMV with antivirals only when a patient with severe colitis is failing to respond to immunosuppressive therapy [1, 3]. The ECCO state that, when detected only by PCR, in the absence of supportive histology or immunohistochemistry, the detection of CMV may not always suggest disease. They recommend the discontinuation of immunosuppressive agents only in cases of severe colitis with detection of CMV in the mucosa but do not state clearly in what form the detection may occur. The British Society of Gastroenterology (BSG) refers to the ECCO guidance but consider the situation in greater detail. They describe the situation in which active CMV colitis in a patient on immunosuppression is difficult to distinguish from a flare of IBD. They suggest treatment of CMV and discontinuation of immunomodulators in severe or refractory colitis in which CMV is detected by histology and PCR [22]. Our proposed flow chart (Fig. 3) illustrates the British approach to management. However, these guidelines may be outdated as more recent data suggest that early detection and treatment of CMV may be beneficial. Roblin et al. found that a CMV DNA load above 250 copies/mg in tissue was predictive of resistance to three successive treatment regimens for ulcerative colitis and suggest that it might be prudent to treat those patients found to have high CMV DNA levels before they deteriorate [23].

## Summary

The current position with respect to the detection of CMV in the bowel mucosa in colitis is unclear. This is particularly important in the patient with severe colitis on immunosuppression and detection of CMV. The risks and benefits of reducing immunosuppression and prescribing treatment for CMV in such a patient are not determined. There is an urgent need for further work in this field. Studies are needed in order to clearly define the significance of a positive CMV in the bowel (or stool) in colitis; to determine if patients with CMV detected by PCR, but not biopsy-proven, would benefit from antiviral treatment; and to determine whether reduction in immunosuppression causes more harm than good in such patients.
